# Association between serum ferritin and liver iron concentration with cardiac iron in pediatric thalassemia major patients

**DOI:** 10.1186/1532-429X-18-S1-P295

**Published:** 2016-01-27

**Authors:** Antonella Meloni, Maddalena Casale, Aldo Filosa, Maria Giovanna Neri, Lorella Pitrolo, Maria Paola Smacchia, Stefania Renne, Antonino Vallone, Vincenzo Positano, Alessia Pepe

**Affiliations:** 1 CMR Unit, Fondazione G. Monasterio CNR-Regione Toscana, Pisa, Italy; 2 grid.413172.2Centro per la Cura delle Microcitemie, Cardarelli Hospital, Napoli, Italy; 3 grid.417108.bEmatologia II con Talassemia, Ospedale "V. Cervello", Palermo, Italy; 4 grid.417007.5Unità Operativa Complessa di ImmunoEmatologia, Policlinico Umberto 1, Roma, Italy; 5 Struttura Complessa di Cardioradiologia-UTIC, P.O. "Giovanni Paolo II", Lamezia Terme (CZ), Italy; 6 Istituto di Radiologia, Az. Osp. "Garibaldi", Presidio Ospedaliero Nesima, Catania, Italy

## Background

Recently, the ability of LIC (liver iron concentration) and serum ferritin in predicting myocardial iron overload (MIO) has been challenged by magnetic Resonance Imaging (MRI) monitoring which demonstrated no or weak correlation between serum ferritin or LIC and MIO. Anyway, the role of this traditional markers could result particularly useful in pediatric population, where MRI assessment is difficult to carry out, because of early age, scarce collaboration or limited availability. So, we derived objective thresholds for these markers for predicting cardiac T2*<20 ms in pediatric patients.

## Methods

From the 2171 patients with hemoglobinopathies enrolled in the MIOT (Myocardial Iron Overload in Thalassemia) network, we retrospectively selected 107 paediatric patients with thalassemia major (TM) (61 boys, median age 14.4 years).

MIO was assessed using a multislice multiecho T2* approach. Hepatic T2* values were assessed in a homogeneous tissue area and converted into LIC.

## Results

Twenty-three patients (21.5%) showed an abnormal global heart T2* value and none of them was under 7.9 years of age.

**Figure 1:**
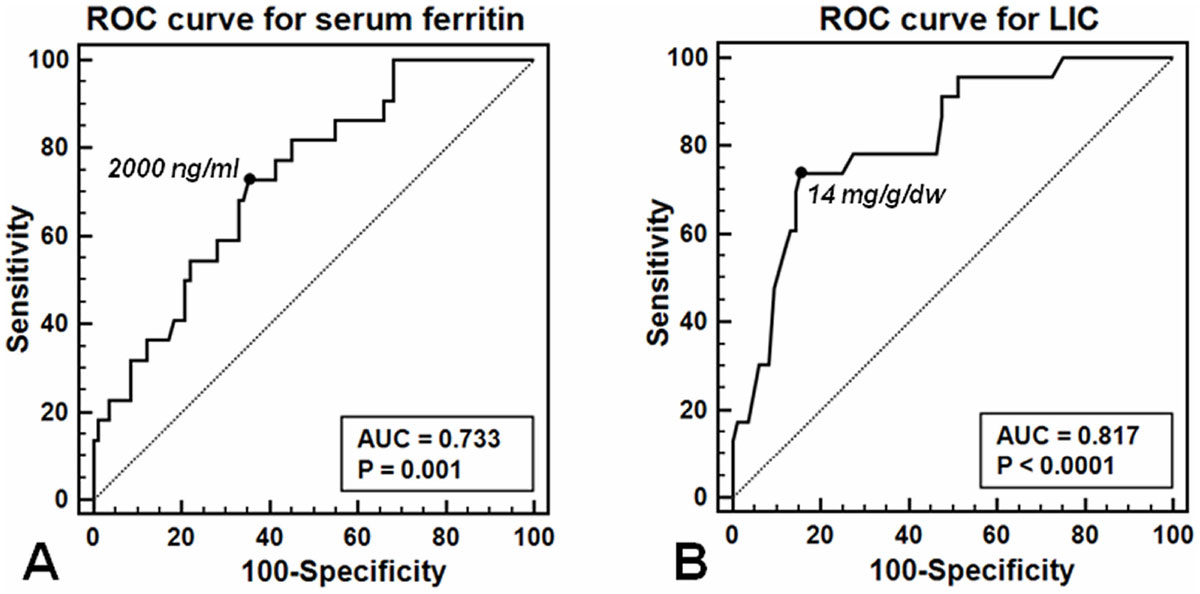
**ROC curves for serum ferritin levels (A) and MRI LIC values (B)**.

Serum ferritin was negatively correlated with global heart T2* values (r=-0.425; p < 0.0001). Using ROC curve analysis, a serum ferritin of 2000 ng/ml was found to be the best threshold for discriminating the presence of cardiac iron with an area under the curve (AUC) of 0.733 (P=0.001) (Figure 1A) (Sensitivity= 0.73 and Specificity=0.65). Odds ratio (OR) for global heart T2* values<20 ms was 4.9 (1.7-13.8 95%CI; P=0.003) for serum ferritin levels≥2000 ng/ml.

There was a significant negative correlation between global heart and MRI LIC values (P=-0.436; p < 0.0001). Using ROC curve analysis, a LIC≥14 mg/g/dw was found to be the best threshold for discriminating the presence of MIO in children with an AUC of 0.817 (p < 0.0001) (Figure 1B) (Sensitivity= 0.74 and Specificity=0.85 ). OR for abnormal global heart T2* values was 30.08 (3.58-252.68 95%CI; P=0.002) for patient with MRI LIC≥14 mg/g/dw versus patients with normal MRI LIC.

## Conclusions

A weak connection between serum ferritin levels or hepatic iron and cardiac iron was demonstrated in our pediatric population. Anyway, MRI LIC≥14 mg/g/dw and serum ferritin levels≥2000 ng/ml were found to be significant risk factors for a global heart T2* value<20 ms in TM children.

